# Using intracellular markers to identify a novel set of surface markers for live cell purification from a heterogeneous hIPSC culture

**DOI:** 10.1038/s41598-018-19291-4

**Published:** 2018-01-16

**Authors:** Elizabeth J. Paik, Alison L. O’Neil, Shi-Yan Ng, Chicheng Sun, Lee L. Rubin

**Affiliations:** 1 Department of Stem Cell and Regenerative Biology, Harvard University, Cambridge, MA 02138 USA; 2 Harvard Stem Cell Institute, Cambridge, MA 02138 USA

## Abstract

Human embryonic stem cells (ESCs) and induced pluripotent stem cells (iPSCs) can provide sources for midbrain dopaminergic (mDA) neural progenitors (NPCs) for cell therapy to treat Parkinson’s disease (PD) patients. However, the well-known line-to-cell line variability in the differentiation capacity of individual cell lines needs to be improved for the success of this therapy. To address this issue, we sought to identify mDA NPC specific cell surface markers for fluorescence activated cell sorting (FACS). Through RNA isolation after sorting for NPCs based on staining for cell-specific transcription factors followed by microarray, we identified two positive cell surface markers (CORIN and CD166) and one negative cell surface marker (CXCR4) for mDA NPC sorting. These three markers can enrich floor plate NPCs to 90% purity, and the sorted NPCs more efficiently differentiate to mature dopaminergic neurons compared to unsorted or CORIN^+^ alone mDA NPCs. This surface marker identification strategy can be used broadly to facilitate isolation of cell subtypes of interest from heterogeneous cultures.

## Introduction

Parkinson’s disease (PD) is characterized by the specific loss of substantia nigra (A9-subtype) mDA neurons, and cell replacement therapy is considered a suitable treatment to replace the lost neurons. However, initial cell transplantation attempts using fetal midbrain sources were compromised by the lack of standardized tissue preparation procedures leading to variable clinical outcomes among transplant recipients^1^. Recent successes with *in vitro* differentiation of mDA neurons from human ESCs and iPSCs have revived the possibility of cell replacement therapy^2^, but the underlying problems of cell heterogeneity and variability still remain. In this study, we present a novel method to identify the cell surface proteome of human iPSC-derived mDA NPCs. Using this method, which involves initial genome-wide profiling of intracellularly-labelled LMX1^+^FOXA2^+^ mDA NPCs, we were able to obtain a population of mDA NPCs with up to 90% purity. This approach is also widely applicable to other cell types of interest where robust intracellular markers exist, but surface antigens for cell purification remain unknown.

## Results

### Midbrain dopaminergic neurons are efficiently generated in suspension culture

**Figure 1:**
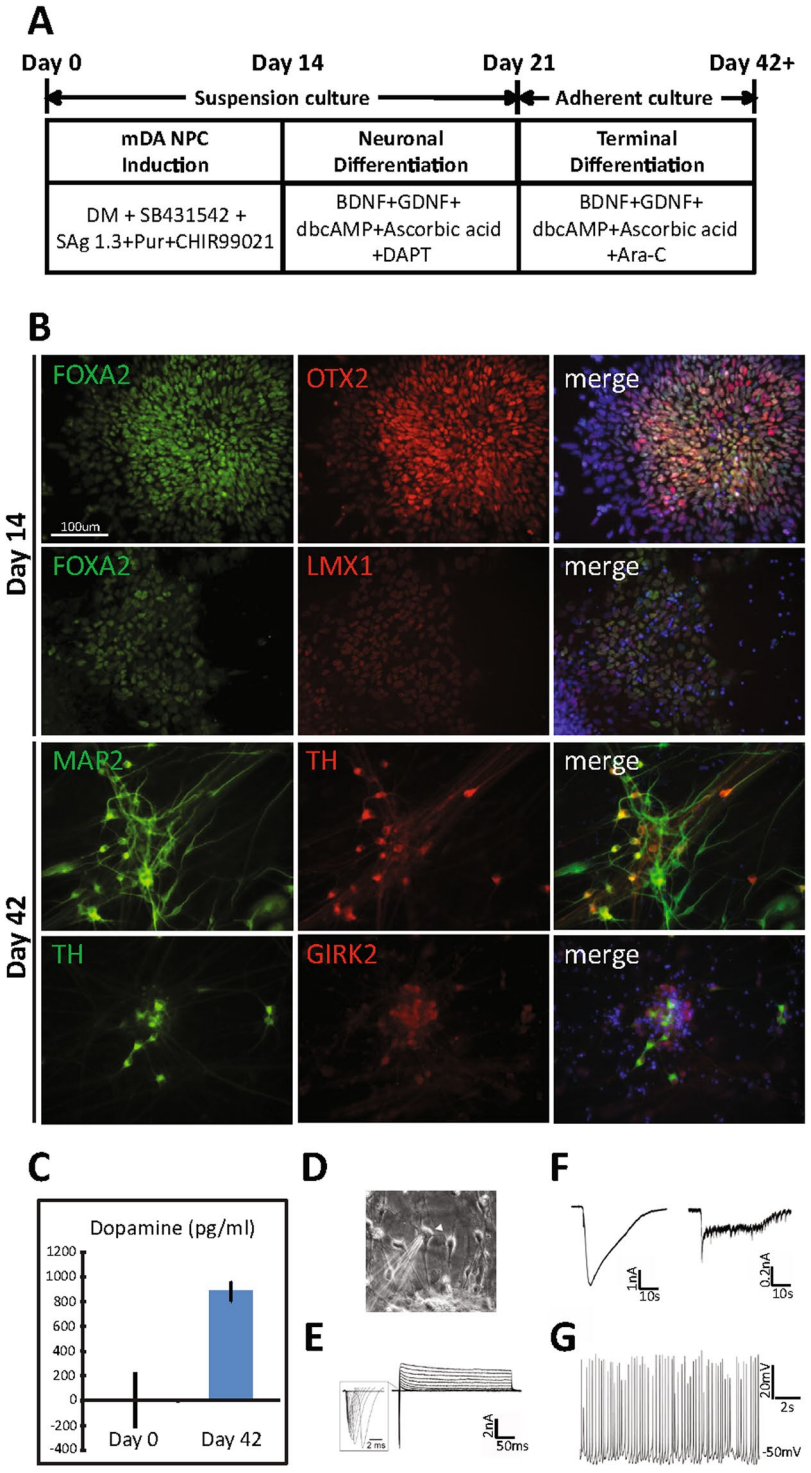
mDA differentiation protocol yields mDA NPCs at day 14 and mDA neurons at day 42. (**A**) mDA differentiation scheme. After dissociation, iPS cells were kept in suspension culture for 21 days. In the first 14 days, cells were induced with DM (Dorsomorphin), SB431542, SAg 1.3 (Smoothened agonist), Pur (Purmorphamine), and CHIR99021. From day 14 through day 21, cells were differentiated in the neuronal differentiation medium containing BDNF, GDNF, dbcAMP, Ascorbic acid, and DAPT. From day 21, cells were further differentiated in the terminal differentiation medium containing BDNF, GDNF, dbcAMP, Ascorbic acid, and Ara-C. (**B**) Immunostaining of day 14 (top two rows) and day 42 (bottom two rows) 18a cells. (**C**) The mean concentration (pg/ml) of dopamine released by day 0 cells and day 42 18a cells. (**D**) Phase contrast image showing human iPSC 18a-derived dopaminergic neuron cultures after 1 month adherent culture. Arrowhead points to a recorded cell. (**E**) Representative traces showing whole-cell voltage-gated Na^+^ and K^+^ currents recorded in human iPSC 18a-derived dopaminergic neuron culture. (**F**) Representative traces showing responses to GABA and AMPA (100 representative traces each) (**G**) Representative traces showing spontaneous action potentials. The resting membrane potential was −50 mV.

To generate mDA NPCs from iPS cells, we optimized two previously published protocols^2,3^ (Fig. 1A). In our modified protocol, by day 14 of the differentiation, the cells expressed the mDA NPC markers, FOXA2, OTX2, and LMX1 (Fig. 1B). On day 21, we saw the co-expression of FOXA2/LMX1 and LMX1/Nurr1 (Supplementary Fig. S1). By day 42, cells expressed mDA neuron markers including TH, EN1 and the A9-subtype marker, GIRK2 (Fig. 1B and Supplementary Fig. S1). This method of differentiation was applied across three wild-type iPSC lines: 1016a, 18a, and BJ-riPS, but yielded different final percentages of TH^+^ neurons at day 40 (1016a 14%, 18a 45%, and BJ-RiPS 19%). Such variability is commonly observed when differentiating neurons from multiple iPSC lines^4^ (Supplementary Fig. S2).

To establish that our cultured mDA cells were functional, the release of dopamine was confirmed using ELISA. In line with previous measurements of derived DA neurons, our cells released 800 pg/ml of dopamine in 48 hour conditioned media^5^ (Fig. 1C). Next, we applied whole-cell patch-clamp recordings to examine the electrophysiological properties of these cells (Fig. 1D-G). All recorded cells (n = 11) showed typical voltage-gated Na^+^ and K^+^ currents (Fig. 1E) and responded to both major inhibitor and excitatory neurotransmitters (n = 5; Fig. 1F). A subset of cells (3 out of 11) fired repetitive action potentials spontaneously (Fig. 1G), which is characteristic of mature DA neurons. Our electrophysiology results are comparable to those obtained by others^6–8^. These results indicate that iPSCs successfully differentiated into mDA neurons with the modified protocol, thereby indicating the presence of functional mDA NPCs in our culture.

### Identification of putative mDA NPC – specific surface markers

**Figure 2:**
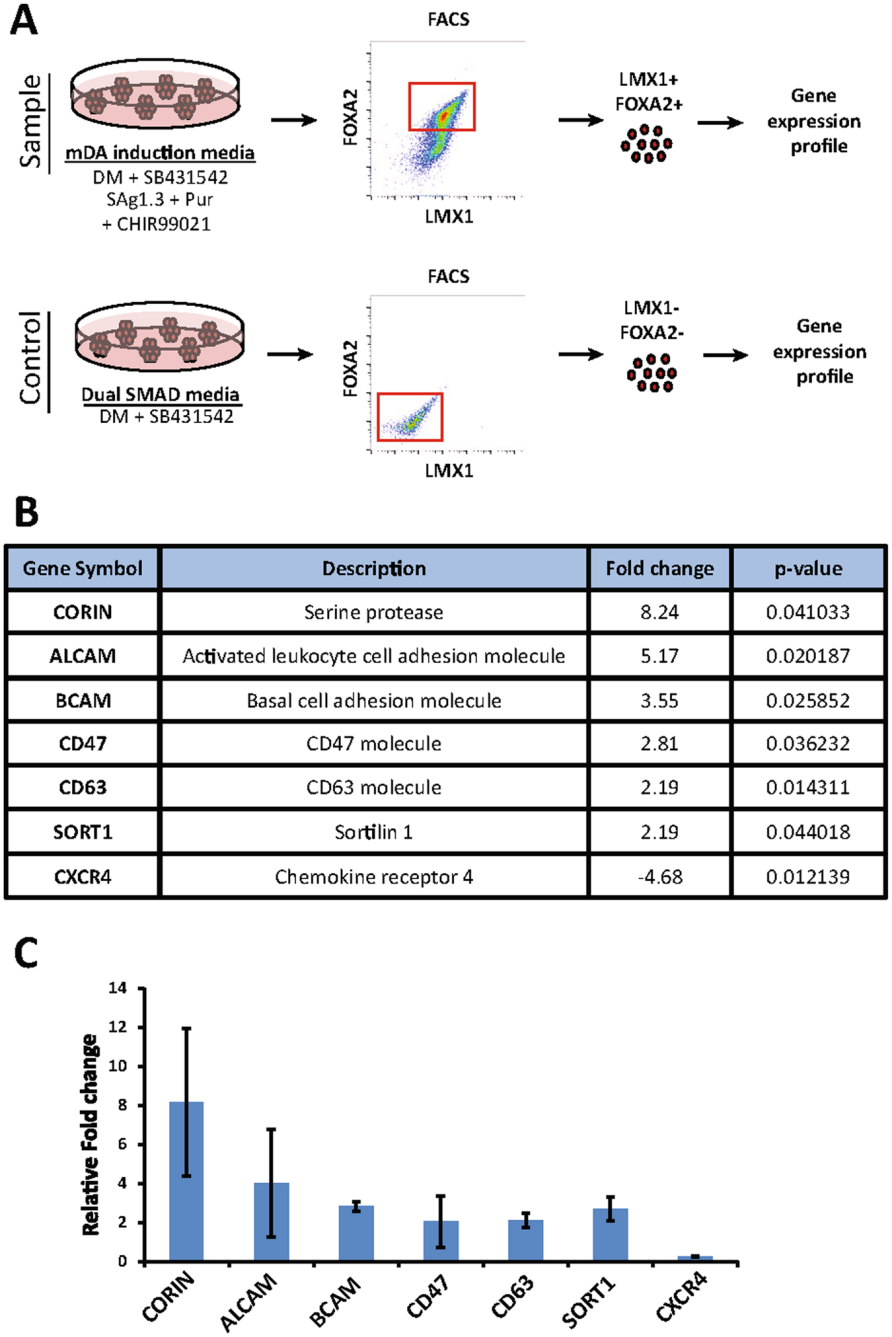
Identification of putative surface markers of mDA NPCs. (**A**) Schematic of method. Using the 18a line, sample (top) was prepared by mDA induction medium for 14 days, and control (bottom) was induced in dual SMAD medium for 14 days. Both groups were incubated with LMX1 and FOXA2 antibodies and FACS purified. The resulting LMX1^+^FOXA2^+^ (sample) and LMX1^-^FOXA2^-^ (control) populations were then processed through microarray to obtain the gene list. (**B**) The list of putative surface marker genes. The detailed list obtained through GO analysis can be found in the supplemental data. (**C**) Confirmation of RNA expression level of putative surface marker genes through qRT-PCR following MARIS. Following the 14 day-differentiation of BJ-RiPS, cells were fixed, stained with LMX1 and FOXA2 antibodies. These cells were then FACS purified into LMX1^-^FOXA2^-^ and LMX1^+^FOXA2^+^ populations. The bar graph shows the relative expression of each putative surface marker gene in the LMX1^+^FOXA2^+^ population compared to the LMX1^-^FOXA2^-^population. (n = 2)

As shown in Fig. 1B, *in vitro* differentiation of iPSCs gave rise to FOXA2^+^ cells by day 14. We noted that there was cell line-to-cell line and batch-to-batch variation in differentiation efficiency, which has also been reported by others (Supplementary Fig. S2)^4^. To enrich our mDA cultures and close the efficiency gap between batches/lines, we chose to search for NPC-specific surface markers to increase the percentage of LMX1^+^FOXA2^+^ cells. We reasoned that these markers might be identified at the mRNA level, and thus we conducted gene expression profiling using a microarray approach. Using the MARIS (Method for Analyzing RNA following Intracellular Sorting) approach for which dissociated single cells were fixed, permeabilized and labelled with antibodies recognizing the transcription factors LMX1 and FOXA2, we eliminated the need to have reporter cell lines^9,10^ (Fig. 2A). Using the 18a iPSC line, we sorted LMX1^+^FOXA2^+^ day 14 cells by MARIS-FACS. To generate the needed large number of LMX1^-^FOXA2^-^ cells, we used dual SMAD inhibition to create a similar (forebrain) pool of progenitors to use as the negative control population (Fig. 1A, Supplementary Fig. S3). Comparison of mRNA expression levels between LMX1^+^FOXA2^+^ and LMX1^-^FOXA2^-^ cells revealed 530 differentially-regulated genes (278 up-regulated, 252 down-regulated) in the LMX1^+^FOXA2^+^ population (fold change > 2.0, false discovery rate < 0.05, Supplemental Table 1). The differentially-regulated gene list was enriched with classical mDA genes such as FOXA1, FOXA2, LMX1a, and LMX1b, confirming the success of this procedure (Supplemental Table 2). We then used Gene Ontology (GO) analysis^11^ to discover a putative surface marker gene list. The GO analysis revealed 41 up-regulated (14.7%) and 26 down-regulated genes (10.3%) that were associated with the plasma membrane (Supplemental Table 2). In the top hits, CORIN and CD47, have been confirmed by others to be expressed in mDA NPCs and  have been used to enrich for mDA NPCs^12,13^. This suggested that our approach using MARIS to identify cell surface markers might be both viable and successful. Because CORIN^+^ sorting alone does not give a pure mDA NPC population^14^, we decided to introduce additional sorting parameters by focusing on other surface markers from our microarray hit list. Based on the fold change seen in the microarray data and the availability of FACS-compatible antibodies, we subsequently validated via qPCR 6 surface markers in addition to CORIN, namely BCAM, CD63, CD47, SORT1, CD166 (ALCAM) and CXCR4 (Fig. 2b and c). These markers were verified by qPCR in a different cell line, BJ-RiPS, than the one used for the microarray, 18a, to confirm that microarray results were not cell line specific.

**Figure 3:**
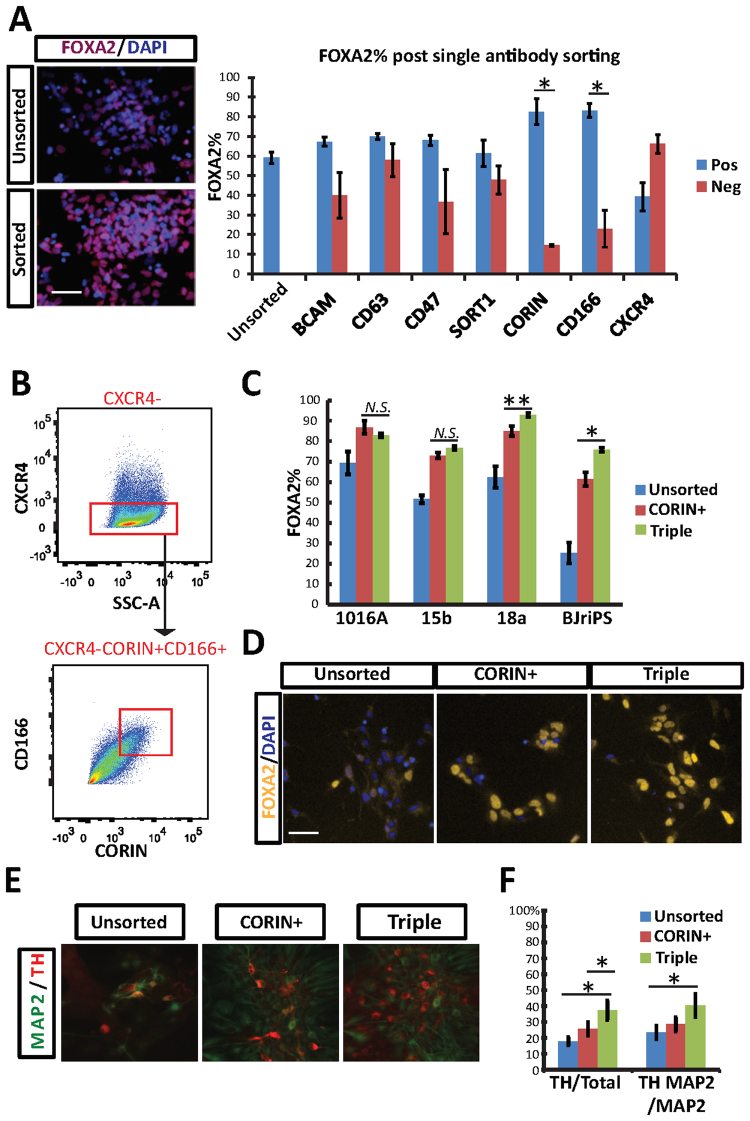
The use of CXCR4^-^CORIN^+^CD166^+^ markers for enrichment of mDA NPCs. (**A**) Identification of mDA NPC surface markers by single antibody sorting. Day 14 BJ-RiPS cells were stained with antibodies corresponding to the putative surface marker genes shown in Fig. 2B. After staining, cells were FACS purified into positive and negative populations, and stained with FOXA2. n = 3, *(p < 0.05). White bar = 50 um. (**B**) CXCR4^-^CORIN^+^CD166^+^ FACS purification scheme. (**C**) FOXA2^+^% quantification comparison between unsorted, CORIN^+^, and triple (CXCR4^-^CORIN^+^CD166^+^) cells from 1016A, 15b, 18a, and BJ-RiPS-derived NPCs. N.S. (Non-significant), *(p < 0.05) and **(p < 0.005) using t-test (n = 3). (**D**) Representative photos of BJ-RiPS-derived NPCs from Fig. 3C. White bar = 50 um. (**E**) Representative photos from *in vitro* differentiation following the FACS purification. The BJ-RiPS cells were fixed at day 42 and stained with antibodies against MAP2, TH, and DAPI. White bar = 50 um. (**F**) Quantification of Fig. 3E. Using t-test, n = 3, *(p < 0.05). *Error bars represent SEM*.

Interestingly, CXCR4 has been shown to be an important surface marker for the proper migration of dopaminergic neurons in mouse models; yet, it is down regulated in our day 14 human iPSC-derived mDA NPCs.^15,16^ To investigate this, we stained iPS cells for CXCR4 and looked at the CXCR4 transcript level over the course of their differentiation. We found by qPCR and immunostaining that the expression level of CXCR4 increased over time (Supplemental Fig. S4). We believe that at day 14 of our differentiation it is a negative marker of NPCs as evidenced by the CXCR4^-^ population being enriched for FOXA2^+^ cells (Fig. 3A) but is re-expressed in mature DA neurons. Additionally, the low expression of CXCR4 in the DA progenitor pool is consistent with published data in the developing mouse brain.^16^

### FACS antibody screening identifies a specific surface marker code for mDA NPCs

To test the ability of our identified putative surface markers to isolate mDA NPCs, we conducted a mini-FACS screen with our 18a mDA NPCs at day 14 of differentiation. The day 14 cells were incubated with antibodies labelling each of the 7 putative surface markers and purified using FACS. After FACS sorting, both the positive and negative populations were plated and counterstained with a FOXA2 antibody (Fig. 3A). This assay showed that CORIN^+^ and CD166^+^ cells, our top two hits from the microarray, had a higher percentage of FOXA2^+^ cells (82% and 83%) compared to the unsorted cells (59%). The CXCR4^-^ population was also enriched with FOXA2^+^ cells (66%), as expected from its low mRNA expression in the day 14 microarray results. On the other hand, two other antibodies (CD47 and SORT1) did not enrich for FOXA2^+^ cells even though these surface markers labelled some cells by FACS (Fig. 3A, Supplementary Fig. S5). The identified markers BCAM and CD63 labeled few cells by FACS (Supplementary Fig. S5). One reason for this is that BCAM, CD63, CD47 and SORT1 had relatively smaller mRNA expression fold changes compared to CORIN and CD166 (Fig. 2B), and such fold change values may translate into insignificant changes at the level of surface protein expression. Additionally, the microarray differences were calculated from a population of control cells (LMX1^-^/FOXA2^-^) that may not exist in large numbers in the starting heterogeneous DA NPC culture.

Next, we assessed whether combinations of CORIN, CD166 and CXCR4 could further enrich for mDA NPCs across multiple iPS-derived NPCs. We examined NPCs derived from four independent wildtype iPSCs, 1016A, 15b, 18a and BJ-RiPS^17–19^. To do this, we stained the day 14 cells with all three antibodies, and purified the CXCR4^-^CORIN^+^CD166^+^ populations (Fig. 3B). We then analyzed the FOXA2 expression of these isolated cells. From this assay, we saw that CXCR4^-^CORIN^+^CD166^+^ cells were enriched in FOXA2^+^ cells across all four iPS-derived NPCs (Fig. 3C). Remarkably, these three antibodies worked well even in the BJ-RiPS cell line that had poor differentiation efficiency (unsorted had only 25% FOXA2 + cells, Fig. 3C). In BJ-RiPS-derived NPCs, the triple antibody combination performed much better compared to CORIN antibody alone in terms of FOXA2^+^% (75% vs. 61%) (Fig. 3C and D).

Finally, we asked whether the sorted day 14 cells can efficiently generate mDA neurons upon further differentiation *in vitro*. To do this, we sorted CXCR4^-^CORIN^+^CD166^+^ NPCs on differentiation day 14, differentiated them in suspension culture for an additional 7 days, then kept them attached in Matrigel-coated plates for 21 days further. At day 42 in the BJ-RiPS, immunostaining showed that CXCR4^-^CORIN^+^CD166^+^ cells more efficiently gave rise to TH^+^ cells (37%) compared to either unsorted (18%) or CORIN^+^ (26%) cells (Fig. 3E and F). The increase in TH^+^% at day 42 was also seen across lines 18a (unsorted 46%, sorted 60%) and 1016a (unsorted 14%, sorted 31%) (Supplementary Fig. S2). This result demonstrates that the specific surface protein profile that we identified marks a more homogeneous pool of mDA NPCs that are capable of making TH^+^MAP2^+^ neurons. We did not identify the TH- population in our cultures; however others have identified them as serotonergic neurons and other non-specific neurons^20^.

## Discussion

### In this report, we have shown the utility of the intracellular labelling approach in identifying surface markers for live cell purification

Using mDA NPCs as a therapeutically relevant example, we demonstrated that CXCR4^-^CORIN^+^CD166^+^ cells from *in vitro* differentiated human iPSCs are enriched for mDA NPCs which further differentiate into mDA neurons *in vitro*. Knowledge of cell surface marker profiles of mDA cells have immediate applicability. Previous Parkinson’s disease clinical trials with fetal midbrain transplantation showed variable results with some patients suffering side effects such as dyskinesia, thought to have been caused by contaminating cell types. In this respect, purification of mDA NPCs using our three surface markers would enhance the possibility of successful transplantation therapy by creating a more homogeneous pool of transplantable cells. This method would also be applicable for identification of surface markers in other neural and non-neural cell types of clinical or research interest.

Even though previous surface marker discovery studies have either used an unbiased FACS antibody screening approach^21^ or used markers that were previously identified in different animal systems^12^, these approaches typically require a genetically-engineered reporter cell line. The primary advantage of our approach is that the need for reporter lines is eliminated due to the frequent knowledge of intracellular proteins, such as transcription factors or other cytoplasmic components, that mark target cell populations. The three surface markers we identified in this study using the MARIS method, CORIN, CD166 and CXCR4, have been previously associated with mDA cells^12,14,22,23^ and a recent microarray study using Ngn2-GFP mice also showed CORIN and CD166 to be expressed in mDA NPCs^23^. These examples show that the MARIS method is a reliable way of identifying putative cell surface markers.

### In conclusion, we have presented an effective method to elucidate the cell surface proteome by MARIS

This has in turn led to the discovery of a unique surface marker combination, CXCR4^-^CORIN^+^CD166^+^, that can be used for mDA NPC purification. This led to the production of a more homogeneous population of mDA NPCs even from iPSC lines that differentiate poorly that can be used for subsequent transplantation experiments. Even though we are not able to identify the type of neuron the TH^-^ pool represents, the enhanced purity of the mDA neurons lends itself to the preparation of *in vitro* assays where the mDA neuron population is the target. While we have not assessed the ability of these sorted cells to differentiate or engraft upon animal transplantation, the successful *in vitro* differentiation shows they are more capable at generating TH^+^ cells than are unsorted cells. It is also reasonable to assume that once implanted, the percentage of TH^+^ neurons from our sorted cultures will be increased through interactions with the midbrain niche. We believe the combination of the three surface markers will highly enrich the mDA neurons derived from human iPSCs, remove uncommitted pluripotent cells, and thus would eliminate the contaminating cells during the future cell transplantation therapy for PD patients.

## Methods

### Culturing iPSCs

BJ-RiPS, 18a, 1016A and 15b cell lines were cultured on Matrigel coated-plates with mTeSR media as described previously^24^.

### *In vitro* differentiation of iPSCs to mDA neurons

iPSCs were dissociated with Accutase and resuspended in neural induction medium containing DMEM/F12, Neurobasal, N2, B27 without vitamin A, Dorsomorphin (1 µM), Purmorphamine (1 µM), SAG 1.3 (1 µM), SB431542 (10 µM), and CHIR99021 (0.5 µM). Cells were then grown in suspension using ultra non-attachment plates. On day 14, cells were switched into neural differentiation medium containing DMEM/F12, Neurobasal, N2, B27 without vitamin A, BDNF (20 ng/ml), GDNF (20 ng/ml), Ascorbic Acid (200 µM), dibutryl-cAMP (0.5 mM), and DAPT (2.5 µM). On day 21, cells were plated on Matrigel-coated plates, and the medium was switched to terminal differentiation medium containing DMEM/F12, Neurobasal, N2, B27 without vitamin A, BDNF, GDNF, Ascorbic Acid, dibutryl-cAMP, and Ara-C (1 µM) until the final dissociation.

For the microarray, the LMX1^+^FOXA2^+^ cells were produced as described above, collecting the cells at day 14. For the LMX1^-^FOXA2^-^ control cells, the neural induction medium only contained the dual SMAD inhibitors Dorsomorphin (1 µM) and SB431542 (10 µM) and the cells were also collected at day 14.

### Antibodies

OTX2 (Abcam cat# ab21990 1:500), LMX1 (Millipore cat# AB10533 1:3000), FOXA2 (Santa Cruz cat# 6554 1:500), TH (Pelfreeze cat# P40101–1 1:1000), GIRK2 (Alomone cat# APC-006 1:100), and MAP2 (Abcam cat# ab11267 1:500) were used for immunostaining following fixation. CORIN (R&D cat# MAB2209, 1:250), CD166 (BD cat# 559263, 1:100), and CXCR4 (BD cat# 555976, 1:200), BCAM (R&D cat# FAB1481P, 1:100), CD63 (Abcam cat# ab18235, 1:100), CD47 (Abcam cat# ab134484, 1:100), SORT1 (Bioss cat# bs-6329R-Cy3, 1:100) were used for FACS.

### Dopamine ELISA

Dopamine ELISA was performed on 48 hour conditioned medium obtained from day 42–50 iPSC-derived neurons following the product guideline (Alpco).

### Electrophysiology

Human 18a iPSC-derived neurons were seeded on coverslips in 24-well plates (200,000 cells per well) and co-cultured with mouse cortical astrocytes (CD-1, Charles River Laboratories) for 1 month. Whole-cell patch clamp recordings were performed using a Multiclamp 700B amplifier and a Digidata 1550 Digitizer (Molecular Devices). Data were collected using pClamp 10 software (Molecular Devices, Sunnyvale, CA), sampled at 10 kHz, and filtered at 1 kHz. External solution contained (in mM): 128 NaCl, 30 glucose, 25 HEPES, 5 KCl, 2 CaCl_2_, and 1 MgCl_2_ (pH 7.3). Patch pipettes were pulled from borosilicate glass using a P-1000 Micropipette Puller (Sutter Instrument). Pipette solution contained (in mM): 147 KCl, 5 Na_2_-phosphocreatine, 2 EGTA, 10 HEPES, and 2 MgATP, 0.3 Na2GTP (pH 7.3). The series resistance was typically 10–20 MΩ. To record voltage-dependent Na^+^ and K^+^ currents, the membrane potential was depolarized from −60 mV to 50 mV in 10 mV increments with a holding potential of −70 mV. Leak current was subtracted using an online P/8 protocol. Spontaneous action potentials were recorded without current injection under current-clamp mode.

### FACS

All FACS experiments were run on a MoFlo Astrios instrument and the data was analysed using the FlowJo software.

### MARIS and Microarray

Whole RNA was extracted from LMX1^+^FOXA2^+^ and LMX1^-^FOXA2^-^ day 14 18a cells using a modification of the MARIS method, as previously established by our laboratory^9,10^. Day 14 cells were dissociated to single cells using Accutase and washed with PBS. Next, the cell pellet was resuspended in fresh 4% paraformaldehyde (made in RNAse-free PBS) at a concentration of 1 mL per 5 × 10^6^ cells and incubated at room temperature for 15 minutes. The fixed cells were then pelleted and washed with RNAse-free PBS. The washed pellet was then resuspended in perm/block buffer (0.5% saponin, 1% BSA, 200 U/mL Superase.In RNAse inhibitor (Life Technologies cat# AM2696), in RNAse-free PBS), in the same volume as used for fixation, with primary antibody and incubated at room temperature for two hours with occasional mixing. The cells were then pelleted and washed twice with RNAse-free PBS. Secondary antibody was added in the same perm/block buffer and incubated in the dark, at room temperature, for 45 minutes. Next, the cells were washed with RNAse-free PBS and then resuspended in PBS with Superase.In (200 U/mL). The cells were then filtered through a 45 µm filter and placed on ice in preparation for FACS.

FACS sorted cells were collected in PBS with Superase.In (500 U/mL). Collected cells were then pelleted and the top layer of supernatant was removed leaving ~250 µL in the tube. To this, 10 µL of Proteinase K (RNAse free, Life Technologies cat# 25530049) was added to each tube and vortexed. The digestion was incubated at 50 °C for 50 minutes. After digestion, 500 µL of Trizol was added to the tubes and they were stored at −80 °C until further processed.

RNA was extracted from the Trizol suspension using chloroform and isopropanol and DNA was digested using DNAse (Life Technologies cat# 18068015) following the manufacturer’s protocol. After Trizol extraction, RNA was processed according to Affymetrix guidelines and hybridized onto Gene Chip Human 2.0 ST arrays.

For the microarray, samples of 18a NPC were made in biological triplicate. The Affymetrix Transcriptome Analysis Console was used to do the differential expression analysis. Using a corrected False Discovery Rate (FDR) of 0.05 and a fold change of more than 2 over the LMX1^-^/FOXA2^-^ sample resulted in 530 differentially expressed genes. The full gene list can be found in the supplementary materials and the dataset can be accessed in the NCBI GEO repository (accession number GSE99139).

### Surface Marker Live FACS

Cells were first dissociated to single cells using Accutase and DNAse (1U/µl) through incubation at 37 °C for 15 minutes. Cells were then resuspended in FACS buffer (5% FBS in PBS), filtered through a 45 µm filter, and incubated with the ratαCORIN primary antibody for 30 minutes. The cells were then washed with FACS buffer and spun down. The pellet was resuspended in FACS buffer with the αrat-Alexa488 secondary antibody, CD166-PE, CXCR4-APC and incubated for another 30 minutes on ice. The cells were then washed with FACS buffer and pelleted. Lastly, the cells were resuspended in FACS buffer and filtered right before FACS sorting.

For *in vitro* differentiation post sorting, the cells were resuspended in differentiation media with rock inhibitor at 300,000 cells/mL and allowed to re-aggregate in suspension using non-adherent plates.

### Data availability

The microarray dataset generated and/or analysed during the current study is available in the NCBI GEO repository (accession number GSE99139). https://www.ncbi.nlm.nih.gov/geo/query/acc.cgi?acc = GSE99139.

## Electronic supplementary material

Supplemental Information
