# Supplementary material for: Using intracellular markers to identify a novel set of surface markers for live cell purification from a heterogeneous hIPSC culture
Source: Sci Rep. 2018 Jan 16;8:804. doi: 10.1038/s41598-018-19291-4 (PMC5770419; doi:10.1038/s41598-018-19291-4)
Supplement: Supplementary file 1 — Supplemental Information [file 41598_2018_19291_MOESM1_ESM.pdf]

Supplementary Information

Using intracellular markers to identify a novel set of surface markers for live cell purification from a heterogeneous hIPSC culture

Elizabeth J. Paik*, Alison O'Neil*, Shi-Yan Ng*, Chicheng Sun, Lee L. Rubin[#]

Department of Stem Cell and Regenerative Biology, Harvard University, Cambridge, MA 02138
Harvard Stem Cell Institute, Cambridge, MA 02138

*Co-first authors
#Correspondence:
Lee L. Rubin
lee_rubin@harvard.edu

Supplementary Table S1: Full list of the differentially expressed genes from comparing LMX1^{+}/FOXA2^{+} to LMX1^{-}/FOXA2^{-} neural progenitor cells. The microarray was done on an Affymetrix Human ST 2.0 Array.

Gene Symbol	Fold Change	Affymetrix Transcript Cluster ID
**LINC00261**	49.72	16917810
**SPON1**	17.93	16722278
**CMTM8**	16.23	16938647
**SERPINF1**	12.92	16829570
**SLIT2**	12.83	16965377
**NTN1**	12.56	16831013
**HEG1**	9.19	16958403
**OR51F2**	9.11	16721212
**MYOF**	8.93	16716590
**CORIN**	8.24	16975671
**SLC7A11**	8.12	16979917
**TTC6**	7.81	16783537
**ADAMTS12**	7.46	16995047
**FREM2**	7.34	16774130
**EFEMP1**	7.19	16897834
**SERPINI1**	7.07	16947715
**FOXA1**	6.27	16792181
**TMEM65**	6.25	17080853
**SALL1**	6.24	16826460
**TSPAN2**	5.73	16691314
**PLCL1**	5.64	16889160
**STOX1**	5.59	16705439
**COLEC12**	5.57	16853399
**SHH**	5.55	17064810
**CRISPLD1**	5.52	17070120
**GALR1**	5.38	16853210
**SULF2**	5.24	16919962
**ALCAM**	5.17	16943548
**PRPS1**	5.15	17106031
**FREM1**	5.09	17092531
**TXLNB**	5.07	17024265
**RYR2**	5.03	16679142
**WNT5A**	5.02	16955197
**CMTM7**	4.79	16938656
**RAI14**	4.71	16983848
**PLEKHA5**	4.66	16748888

**PLXDC2**	4.5	16703036
**CCND2**	4.45	16746992
**TTR**	4.38	16851786
**NID1**	4.21	16700888
**DOCK10**	4.18	16909081
**IQGAP2**	4.17	16986351
**ADAMTS9**	4.14	16955822
**GJA1**	4.14	17012148
**RAB3B**	4.03	16687123
**CFC1**	4	16902847
**SEMA3E**	3.98	17059292
**ARMC3**	3.95	16703182
**PTCH1**	3.9	17096091
**TRAM2**	3.82	17020044
**EFNA5**	3.77	16998682
**TUBB4A**	3.74	16867756
**HTR2C**	3.69	17106314
**TCF7L2**	3.64	16709333
**SLC2A13**	3.62	16763195
**FBN1**	3.58	16808793
**BCAM**	3.55	16863074
**LRP2**	3.53	16904827
**PDGFD**	3.52	16743816
**SLIT3**	3.52	17002612
**FJX1**	3.43	16723662
**SYTL5**	3.41	17102566
**CADPS**	3.4	16955699
**GRIN2A**	3.37	16823799
**QKI**	3.37	17014562
**SSFA2**	3.35	16888317
**HHIP**	3.35	16971102
**SHC3**	3.32	17095566
**WLS**	3.31	16688339
**TSPAN6**	3.31	17112607
**GPR98**	3.3	16987140
**DSP**	3.3	17004612
**IGFBP5**	3.29	16908197
**CTGF**	3.23	17023646
**HECW2**	3.22	16906749
**RGS9**	3.2	16837101
**SLC44A1**	3.13	17087790

**PPP1R14C**	3.09	17013664
**FAT3**	3.07	16729986
**USP2**	3.06	16745343
**OTX2**	3.05	16793329
**PDZRN3**	3.04	16956285
**CALU**	3.04	17051236
**RAB11FIP4**	3.03	16833000
**RALGPS2**	3.01	16674292
**LRIG3**	2.98	16766822
**SEPT3**	2.98	16930811
**DTNA**	2.93	16851866
**FAM198B**	2.93	16980974
**TMTC2**	2.92	16754808
**NPC2**	2.91	16794705
**PAPSS2**	2.9	16707009
**LOC151657**	2.9	16957089
**C17orf58**	2.89	16848062
**ATXN1**	2.88	17015862
**MMP2**	2.84	16819064
**SERINC5**	2.84	16997615
**RFX4**	2.82	16756370
**LIX1**	2.82	16998411
**CD47**	2.81	16957095
**RNF220**	2.79	16663899
**PTPRM**	2.75	16850759
**LRRN2**	2.74	16698407
**TSPAN7**	2.73	17102607
**ACAA2**	2.71	16855184
**LDB2**	2.7	16974626
**ST6GALNAC3**	2.69	16666326
**CADPS2**	2.69	17062321
**CAPN6**	2.66	17113362
**EEF1G**	2.65	17063066
**ATP10D**	2.58	16966514
**GPC3**	2.58	17114288
**DGKB**	2.57	17055390
**SLIT1**	2.56	16717032
**CPAMD8**	2.55	16870087
**TMEM63A**	2.53	16699932
**CMTM6**	2.53	16951917
**TEX15**	2.53	17076103

**PXDC1**	2.51	17015207
**BICC1**	2.49	16705089
**ITPR1**	2.49	16936947
**PPIL6**	2.49	17022406
**ECE1**	2.48	16682989
**ATP6AP2**	2.47	17102668
**GSR**	2.46	17076063
**C1QBP**	2.45	16840284
**PON2**	2.45	17059932
**CETN2**	2.45	17115077
**ERMP1**	2.44	17092208
**GALNT2**	2.43	16678579
**PLTP**	2.43	16919703
**ULBP3**	2.43	17024710
**GDE1**	2.42	16824555
**CPD**	2.42	16832768
**CACNG7**	2.42	16865153
**DNAH7**	2.41	16906656
**AJUBA**	2.4	16790614
**KLF3**	2.39	16966008
**PSAP**	2.38	16715241
**ZC3HAV1**	2.38	17063428
**CPNE2**	2.36	16819430
**TANC2**	2.36	16836868
**UGDH**	2.36	16975310
**PTGFRN**	2.35	16669180
**KCNMA1**	2.35	16715793
**RFX2**	2.35	16867613
**HEPH**	2.35	17104283
**ACSS3**	2.34	16754729
**SPTSSB**	2.34	16961056
**NME5**	2.34	17000342
**CCDC47**	2.33	16847605
**HIPK2**	2.33	17063461
**LRRC6**	2.33	17081323
**HTRA1**	2.32	16710126
**GAA**	2.32	16838509
**ZFP36L2**	2.32	16897026
**ITGB8**	2.32	17043982
**PPP4C**	2.31	16817811
**KIAA1407**	2.31	16957583

**LPHN3**	2.31	16967202
**SDC2**	2.31	17071144
**MLEC**	2.3	16757969
**FZD2**	2.28	16834785
**NUP210**	2.28	16950932
**MIR218-1**	2.28	16965432
**PTN**	2.28	17063254
**NFE2L1**	2.27	16835374
**GALNT1**	2.27	16851933
**SEPT10**	2.27	16901593
**LAPTM4B**	2.27	17071208
**GANAB**	2.26	16739396
**MORN2**	2.26	16879276
**DDOST**	2.25	16682842
**MANBA**	2.25	16978444
**CELSR1**	2.24	16936097
**GRSF1**	2.24	16976655
**SPAG6**	2.23	16703150
**SULF1**	2.23	17069816
**WDR96**	2.22	16718184
**RPA1**	2.21	16829580
**SORT1**	2.19	16690566
**CD63**	2.19	16765744
**HSDL1**	2.18	16828762
**ADAMTS16**	2.18	16982870
**STON2**	2.17	16795368
**PCTP**	2.17	16836277
**VGLL3**	2.17	16956532
**CAP2**	2.17	17005138
**SPARC**	2.15	17001927
**NCAN**	2.14	16859969
**DAB2**	2.14	16995645
**ARRDC4**	2.13	16805474
**MOB1B**	2.13	16967602
**PTPN13**	2.13	16968529
**FAT1**	2.13	16982269
**TTC18**	2.12	16715434
**DNAAF1**	2.12	16821437
**RNF157**	2.12	16849148
**CDH11**	2.1	16827041
**CACNG4**	2.1	16837172

**C20orf3**	2.1	16918011
**TWSG1**	2.09	16850896
**RAD23B**	2.09	17087933
**NR6A1**	2.09	17098305
**BCOR**	2.09	17110148
**MACF1**	2.08	16662755
**ALDH18A1**	2.08	16716846
**PRDX3**	2.08	16718922
**ARPP19**	2.08	16809506
**RASGRF2**	2.08	16986777
**CHRDL1**	2.07	17113346
**SEL1L**	2.06	16795394
**HYOU1**	2.05	16745186
**VASN**	2.05	16815509
**MED12L**	2.05	16946966
**TJP2**	2.05	17085685
**ENKUR**	2.04	16712587
**TM9SF3**	2.04	16716983
**OPCML**	2.04	16746290
**UNC119B**	2.04	16757979
**CANX**	2.04	16993397
**DNAH5**	2.04	16994434
**PODXL**	2.04	17062985
**TRAM1**	2.04	17078102
**BASP1**	2.03	16983451
**FRRS1L**	2.03	17096958
**LOC339535**	2.02	16701007
**HYDIN**	2.02	16827874
**LPCAT1**	2.02	16994002
**SALL2**	2.01	16790413
**NPHP1**	2.01	16901632
**PDGFRA**	2.01	16966809
**HNRNPA1**	-2.01	16751993
**SLITRK1**	-2.01	16780092
**SNORD115-20**	-2.01	16798341
**COL11A1**	-2.02	16690211
**DCX**	-2.05	17113378
**VAMP1**	-2.07	16760393
**ARL4D**	-2.08	16834621
**MIR4525**	-2.09	16850286
**CNR1**	-2.1	17021504

**L1TD1**	-2.11	16665439
**H3F3B**	-2.12	16848888
**DNER**	-2.13	16909319
**NAV1**	-2.15	16675794
**DNAJB1**	-2.16	16869653
**SNORD115-44**	-2.19	16798432
**TCTA**	-2.19	16940693
**CA2**	-2.2	17070456
**HIST2H2BC**	-2.22	16692624
**RYR3**	-2.22	16798965
**PCDH15**	-2.23	16714433
**SNORD115-32**	-2.24	16798408
**DUX2**	-2.27	16973271
**GLUL**	-2.28	16696979
**MIR4294**	-2.28	16714084
**CDKN1A**	-2.28	17008007
**ERBB3**	-2.29	16752397
**IER5L**	-2.29	17099027
**ZC3H10**	-2.3	16752485
**RHOB**	-2.3	16877555
**DUX2**	-2.31	16973292
**RGS16**	-2.35	16697018
**ZIC1**	-2.35	16946666
**HIST1H3F**	-2.36	17016400
**SERTAD1**	-2.37	16872443
**ZIC5**	-2.39	16780632
**FOS**	-2.41	16786587
**HIST1H2BC**	-2.42	17005603
**EGFEM1P**	-2.44	16947759
**REXO1L2P**	-2.46	17070478
**KLF7-IT1**	-2.49	16907641
**RNU1-13P**	-2.5	16691877
**ROR1**	-2.53	16665588
**P2RX3**	-2.54	16724818
**SNAR-B1**	-2.56	16874508
**ANKRD37**	-2.58	16972950
**SNORA21**	-2.6	16844046
**NRP2**	-2.64	16889879
**NRN1**	-2.68	17015324
**REXO1L2P**	-2.68	17070480
**POU4F1**	-2.69	16779990

**SNORD4B**	-2.7	16832568
**EPHA7**	-2.7	17021792
**RELN**	-2.71	17061298
**HEXIM1**	-2.74	16834921
**RPS27**	-2.77	16671325
**SNORA42**	-2.79	16694357
**LOC100507303**	-2.79	16823043
**DUSP1**	-2.79	17002846
**LRRC7**	-2.81	16665976
**ZNF878**	-2.89	16869060
**ROBO2**	-2.89	16942743
**UNC5C**	-2.9	16978054
**MIR3189**	-2.98	16859800
**ELAVL2**	-2.99	17092918
**RNU11**	-3.02	16661646
**CNTN2**	-3.03	16676405
**SLC2A3**	-3.03	16760868
**EYA2**	-3.05	16914478
**RASL11B**	-3.05	16966733
**IER5**	-3.06	16674618
**MIR219-2**	-3.08	17098866
**NFASC**	-3.09	16676355
**SNAI1**	-3.09	16914791
**PRDM12**	-3.13	17090350
**SNORA45**	-3.15	16721732
**SGK1**	-3.21	17023810
**DDIT4**	-3.24	16705961
**LOC100132966**	-3.26	16691721
**NRG1**	-3.3	17067696
**SNORA64**	-3.31	16822919
**PCDH8**	-3.35	16779486
**SNAR-E**	-3.37	16873645
**MIR425**	-3.37	16953809
**DPYD**	-3.41	16689969
**STMN2**	-3.44	17070249
**HIST1H2AK**	-3.45	17016496
**BOC**	-3.56	16944010
**EBF1**	-3.56	17002278
**RPLP1**	-3.6	16802553
**SNORA71B**	-3.64	16919223
**HIST2H4B**	-3.7	17005790

**HSPA1B**	-3.71	17030833
**STARD5**	-3.75	16812406
**HIST1H2BN**	-3.75	17005871
**SMOC1**	-3.82	16785897
**SNORA54**	-3.83	16734524
**STC1**	-3.84	17075553
**HSPA1B**	-3.87	17028007
**MIR378D2**	-3.89	17079181
**MEIS2**	-3.93	16807139
**HSPA1B**	-3.94	17026318
**HSPA1B**	-3.94	17027994
**HSPA1B**	-3.94	17030820
**SNHG12**	-4	16684178
**HIST2H2BF**	-4	16692603
**NHLH1**	-4.01	16672630
**FTH1**	-4.04	16739208
**NABP1**	-4.1	16888963
**HIST2H4B**	-4.15	16670377
**JUN**	-4.15	16687875
**HIST2H4B**	-4.15	16692626
**HSPA1B**	-4.19	17038297
**EBF2**	-4.21	17075628
**ZIC2**	-4.22	16776103
**SNORA74B**	-4.23	16992465
**SCARNA27**	-4.23	17015472
**NPPC**	-4.24	16909551
**HSPA1B**	-4.24	17006881
**ADRB2**	-4.31	16990848
**HSPA1A**	-4.41	17006863
**SNORD75**	-4.47	16696533
**SCARNA11**	-4.49	16760514
**GLI3**	-4.53	17056992
**SST**	-4.61	16962574
**HSPA1B**	-4.66	17038309
**CXCR4**	-4.68	16903140
**EBF3**	-4.83	16719562
**METTL12**	-4.85	16725901
**HIST1H3J**	-5.09	17016509
**SNAI2**	-5.1	17077004
**PLK2**	-5.41	16996433
**REXO1L1**	-6.07	17070482

**PMAIP1**	-6.21	16852683
**GADD45B**	-6.35	16856803
**PCDH9**	-6.42	16779667
**MIR3193**	-7.35	16912368
**HIST1H3A**	-8.06	17005532
**HIST1H2BD**	-9.23	17005573
**LOC145845**	-9.38	16807125
**TFAP2B**	-9.67	17009545
**ID1**	-9.68	16912362
**ROCK1P1**	-10.33	16850428
**MIR3143**	-11.2	17005797
**HIST2H4B**	-11.79	17005586
**HIST1H2AH**	-12.6	17005787
**HIST1H2BG**	-13.95	17016390
**HIST1H3H**	-14.12	17005862
**PAX3**	-17.44	16908928
**HIST2H3D**	-21.24	16692614

Dopaminergic Neuron Markers

Gene	fold change
FOXA2	4.99
FOXA1	6.27
LMX1A	1.97
LMX1B	1.34
OTX2	3.05
WNT5A	5.02
SHH	5.55
PTCH1	3.9

Putative Cell Surface Markers

Gene	fold change
CORIN	+8.24
CD166	+5.17
CD239 (BCAM)	+3.55
CD47	+2.81
SORT1	+2.19
CD63	+2.19
CNR1	-2.1
EPHA7	-2.7
PCDH8	-3.35
ADRB2	-4.31
CXCR4	-4.68
PCDH9	-6.42

Supplementary Table S2. Microarray analysis confirmed the fixed, sorted population was of the dopaminergic lineage by revealing upregulation of characteristic genes. Additionally, through the use of GO annotation, the microarray yielded a putative list of positive and negative surface markers for live cell purification.

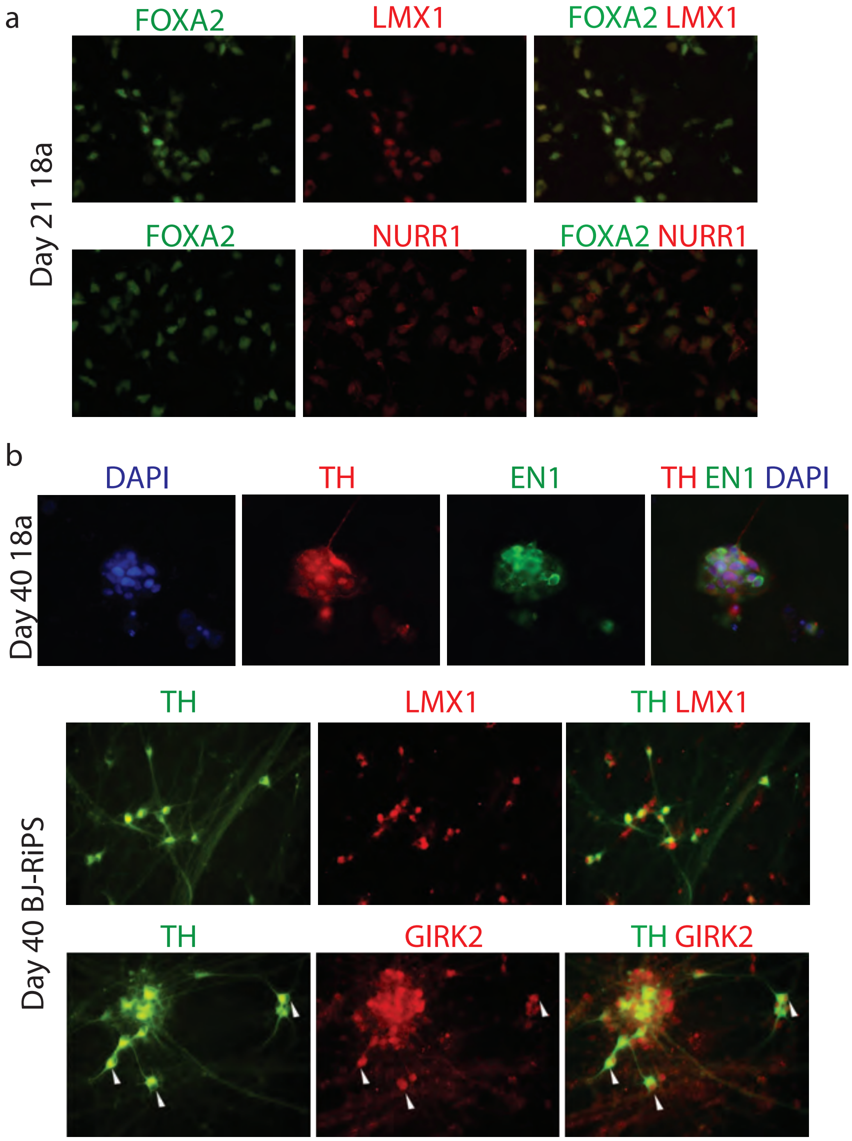

Figure S1. Additional immunostaining characterizing the iPS derived DA neurons. a. Differentiation day 21 18a cells show co-expression of LMX1/FOXA2 and FOXA2/NURR1. b. Differentiation day 40 18a cells show co-expression of TH and EN1 while day 40 BJ-RiPS show co-expression TH/LMX1 and TH/GIRK2.

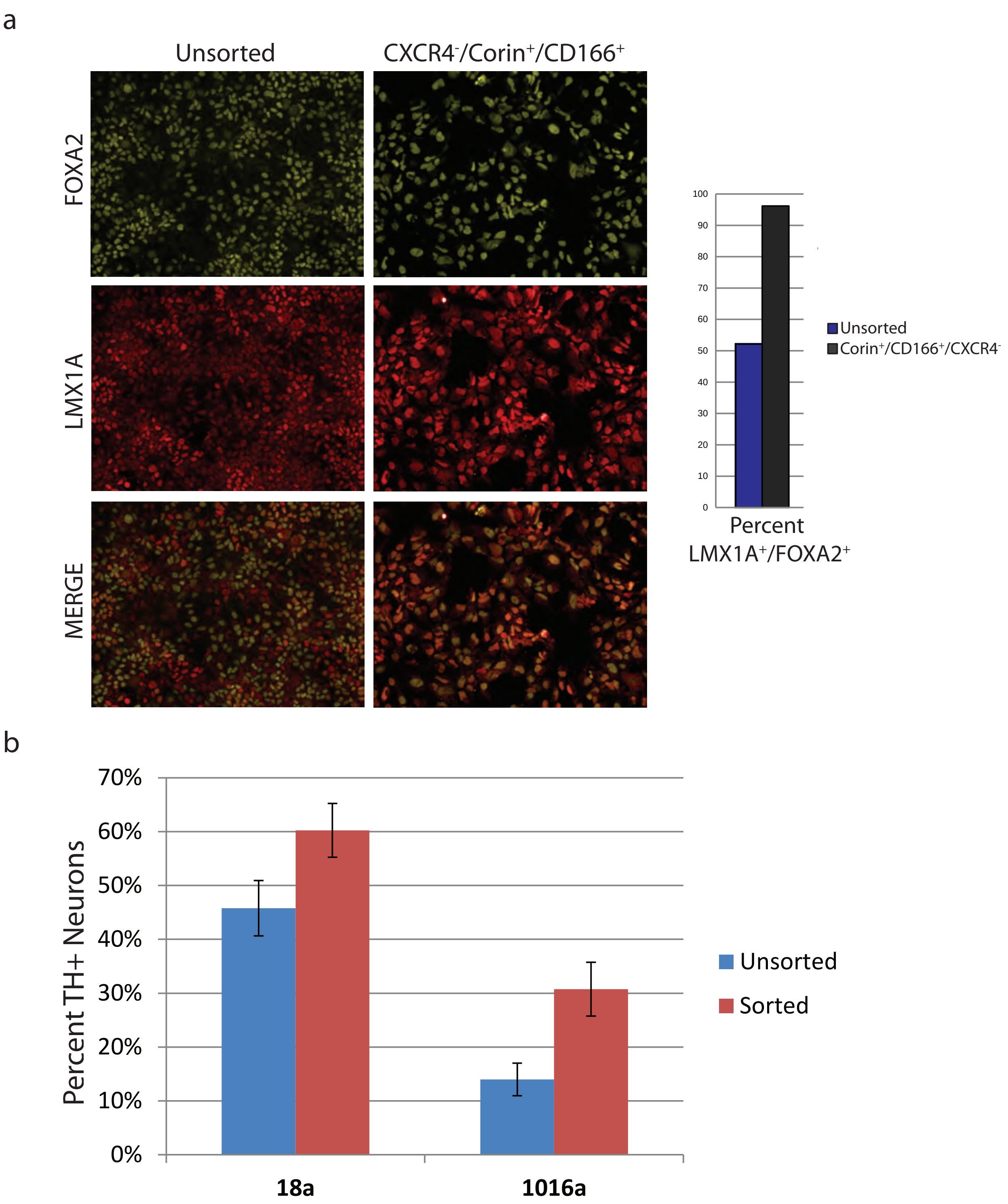

Figure S2. CXCR4⁻/CORIN⁺/CD166⁺ FACS sorting enriches for the FOXA2/LMX1A double positive population and increases the differentiation efficiency to TH+ neurons. a. Day 14 BJ riPS dopaminergic progenitors were live FACS sorted for $CXCR4^-/CORIN^+/CD166^+$ and replated for 48 hours. They were then fixed and stained for FOXA2 and LMX1A. After sorting, the $FOXA2^+/LMX1A^+$ population is highly enriched (n=1). b. The 18a and 1016a cell lines both show increased percentages of TH+ neurons after being sorted as progenitors. n=2, error bars represent 1 standard deviation.

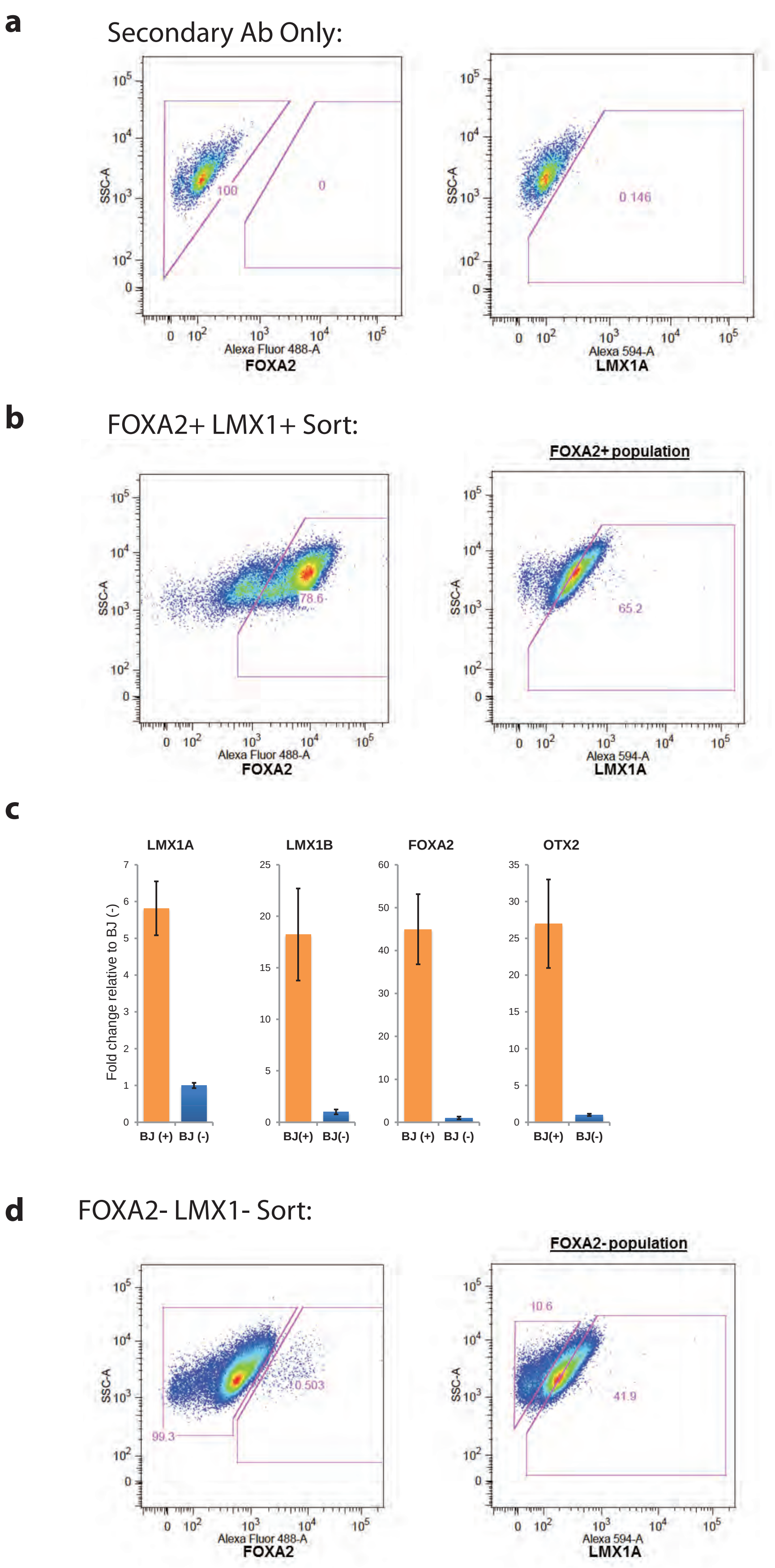

Figure S3. Representative FACS plots for collecting the LMX1+FOXA2+ and LMX1-FOXA2- populations used for microarray analysis.

a. Secondary only control plots used to set gates. b. Representative FACS plots for the LMX1A+FOXA2+ population. c. qPCR of the FOXA2+LMX1A+ sorted population (BJ+) compared to the FOXA2-LMX1A- poulation (BJ-) showing enrichment of dopaminergic progenitor markers LMX1A/B, FOXA2, and OTX2. Error bars represent standard error, qPCR normalized to GAPDH expression, n=3, performed in the BJriPS line. d. Representative FACS plots for the LMX1-FOXA2- population.

a

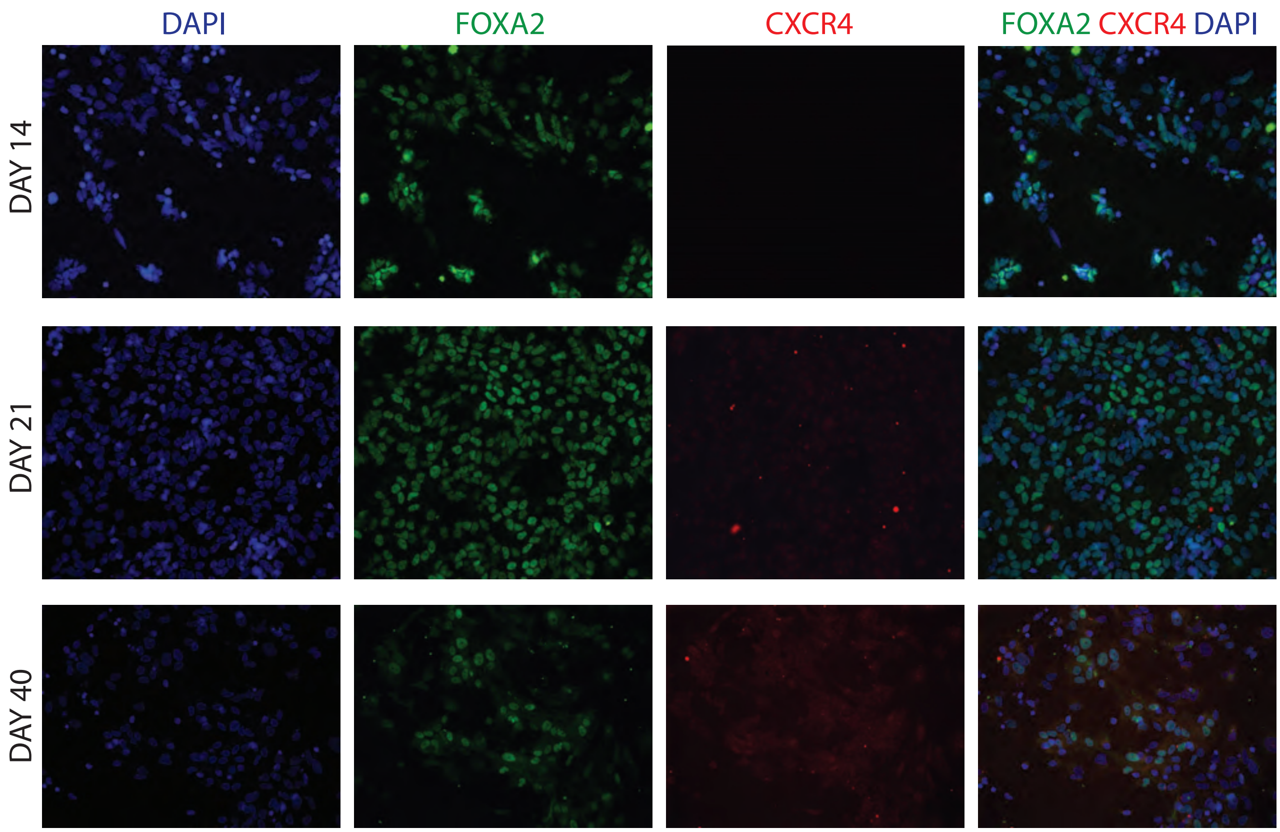

b

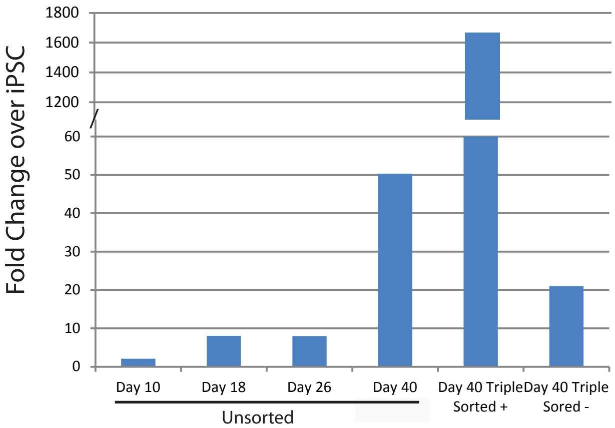

Figure S4. CXCR4 expression is low in mDA NPCs and increases over time.
a. Immunostaining of day 14, 21, and 40 BJ-RiPS differentiation. CXCR4 staining is absent in day 14 but increases to day 40. b. qPCR comparing a differentiation timeline of BJ-RiPS to the starting iPSC shows that CXCR4 expression increases over time. Additionally at day 40 the neurons derived from the CXCR4-CORIN+CD166+ NPCs, had a very high level of CXCR4 expression further illustrating their maturity and higher percentage of mDA neurons over

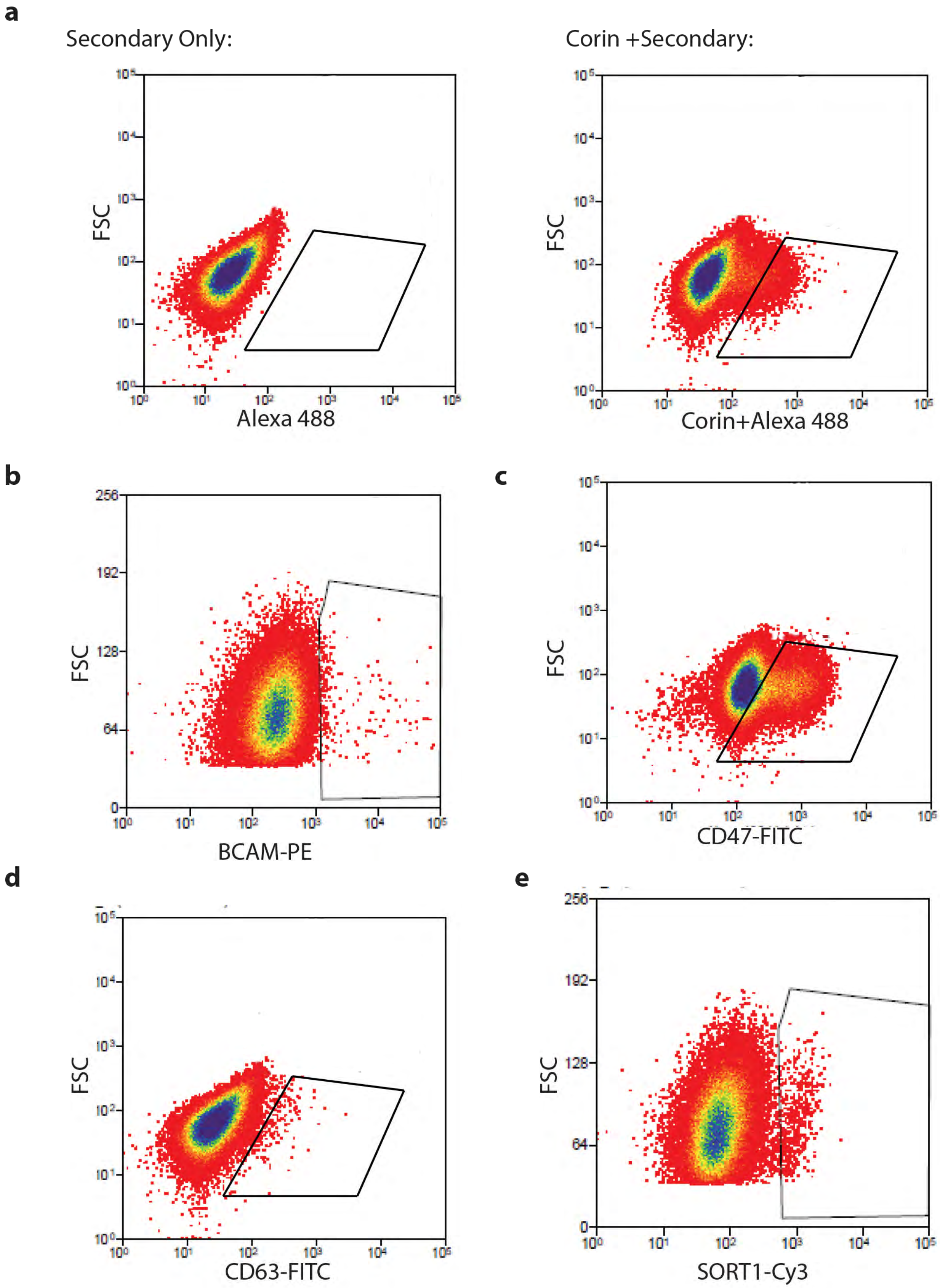

Figure S5. Representative FACS plots of secondary only control and single surface markers that did not enrich for FOXA2+ NPCs. a. Alexa 488 secondary only control (left) and with primary antibody Corin (right). b-e. FACS plots showing that the single surface markers BCAM, CD47, CD63, and SORT1 did yield labelled populations.
